# Antibiotic resistance in porcine pathogenic bacteria and relation to antibiotic usage

**DOI:** 10.1186/s12917-019-2162-8

**Published:** 2019-12-11

**Authors:** I. Holmer, C. M. Salomonsen, S. E. Jorsal, L. B. Astrup, V. F. Jensen, B. Borck Høg, K. Pedersen

**Affiliations:** 1 Technical University of Denmark, Kemitorvet Building 202, Anker Engelunds Vej 1, DK-2800 Kgs. Lyngby, Denmark; 2 SEGES, Agro Food Park 15, DK-8200 Aarhus N, Denmark; 3 National Veterinary Institute, Ulls väg 2B, 751 89 Uppsala, Sweden

**Keywords:** Pig, Antimicrobial resistance, *E. coli*, *Streptococcus suis*, *Actinobacillus pleuropneumoniae*, *Staphylococcus hyicus*

## Abstract

**Background:**

Optimal treatment and prudent use of antimicrobials for pigs is imperative to secure animal health and prevent development of critical resistance. An important step in this one-health context is to monitor resistance patterns of important animal pathogens. The aim of this study was to investigate the antimicrobial resistance patterns of five major pathogens in Danish pigs during a period from 2004 to 2017 and elucidate any developments or associations between resistance and usage of antibiotics.

**Results:**

The minimum inhibitory concentration (MIC) for *Escherichia coli, Actinobacillus pleuropneumoniae, Streptococcus suis, Bordetella bronchiseptica,* and *Staphylococcus hyicus* was determined to representatives of antibiotic classes relevant for treatment or surveillance. *Escherichia coli* isolates were mostly sensitive to fluoroquinolones and colistin, whereas high levels of resistance were observed to ampicillin, spectinomycin, streptomycin, sulfonamides and tetracycline. While resistance levels to most compounds remained relatively stable during the period, resistance to florfenicol increased from 2.1% in 2004 to 18.1% in 2017, likely in response to a concurrent increase in usage. A temporal association between resistance and usage was also observed for neomycin. *E. coli* serovars O138 and O149 were generally more resistant than O139. For *A. pleuropneumoniae*, the resistance pattern was homogenous and predictable throughout the study period, displaying high MIC values only to erythromycin whereas almost all isolates were susceptible to all other compounds. Most *S. suis* isolates were sensitive to penicillin whereas high resistance levels to erythromycin and tetracycline were recorded, and resistance to erythromycin and trimethoprim increasing over time. For *S. hyicus,* sensitivity to the majority of the antimicrobials tested was observed. However, penicillin resistance was recorded in 69.4–88.9% of the isolates. All *B. bronchiseptica* isolates were resistant to ampicillin, whereas all but two isolates were sensitive to florfenicol. The data obtained have served as background for a recent formulation of evidence-based treatment guidelines for pigs.

**Conclusions:**

Antibiotic resistance varied for some pathogens over time and in response to usage. Resistance to critically important compounds was low. The results emphasize the need for continuous surveillance of resistance patterns also in pig pathogenic bacteria.

## Background

The Danish pig industry currently produces approximately 32 million pigs annually [1] (https://agricultureandfood.dk/danish-agriculture-and-food/danish-pig-meat-industry) and in this large production, a wide range of pathogenic bacteria are causing infectious diseases. Among the most prevalent pathogens associated with porcine diseases are *Escherichia coli* (causing diarrhea, oedema disease and septicemia), *Actinobacillus pleuropneumoniae* (causing porcine pleuropneumonia), *Streptococcus suis* (causing e.g. meningitis, arthritis, pneumonia, and septicemia), *Staphylococcus hyicus* (causing exudative epidermitis), and *Bordetella bronchiseptica* (involved in atrophic rhinitis and bronchopneumonia) [2–5]. However, *S. suis* is also a potential zoonotic pathogen and may cause severe infections in humans, such as septicemia, meningitis, permanent hearing loss, endocarditis, and arthritis. The human infections seem to be transmitted by direct contact as it is most often pig farmers, abattoir workers, meat inspectors, butchers, and veterinarians who are affected.

Antibiotics of several classes are widely used for treatment and metaphylaxis of infectious diseases in animals. Development of antibiotic resistance and measures to combat antibiotic resistance have become important issues. It has become very clear that this needs to be addressed in a one-health perspective and strategies and action plans have been adopted to address antibiotic resistance at both national and international level [6, 7]. The one-health approach is necessary as antibiotic resistance and resistant bacteria in humans, food, environment, and animals are connected vessels, where exchange may continuously take place. Therefore, the challenge of antibiotic resistance needs to be addressed not just in animals or in humans, but in all contexts, and the choice of antibiotics for treatment of pigs has a wider perspective reaching beyond the pen. The primary driver for selection and progression of antimicrobial resistance is usage of antimicrobials and there is a connection between usage and resistance although these connections are not always direct and simple [8–11]. The extensive and improper use of antibiotics in both human and veterinary medicine is being recognized as a main selective pressure driving the accelerated emergence and spread of bacterial resistance worldwide [10, 12]. Prudent use of antibiotics for animals is imperative to be able to treat diseased animals as well as humans in the future. Critically important compounds such as 3rd and 4th generation cephalosporin or fluoroquinolones should not be used for animals, and routines that avoid prophylactic use of antibiotics in animal production should be installed. The need for using antibiotics should be reduced through improved animal health, use of vaccines, biosecurity measures, etc. Treatment guidelines may be important decision support tools for veterinarians in their choice of treatment [13]. Such treatment guidelines must be based on scientific knowledge of resistance patterns for causative agents as well as knowledge of the significance of the resistance patterns for treatment of human infections. Therefore, surveillance programs of development of antibiotic resistance for the major veterinary pathogens are important. In Denmark, several initiatives have been taken to reduce the risk of developing antimicrobial resistance. Thus, the use of antibiotics in animals and humans has been monitored by the DANMAP program since 1995 and the program has been refined over the years to include not just usage in kg active compound, but also defined animal daily doses (DADD), thus enabling comparison between species (www.DANMAP.org). All usage of antimicrobials for animals is recorded in the database VETSTAT with information on animal species and quantity together with herd registration number and prescribing veterinarian (https://www.foedevarestyrelsen.dk/Leksikon/Sider/VetStat.aspx).

Historically, there was an increase in the use of antibiotics for pigs in Denmark from 2004 to 2009, followed by a decrease in 2010 and 2011. The reduction during this period was considered to be a result of the “yellow card initiative”, which enforces legal actions on pig farmers who use too high amounts of antibiotics per pig compared to threshold values [14]. During 2016 and 2017, the antibiotic usage for pigs was further reduced by 5 and 4%, respectively, demonstrating the influence of the national control initiative to reduce consumption, and further targets for reduction have already been set [14, 15].

The goal of this reduction is a concurrent reduction in antibiotic resistance. Unfortunately, there is little knowledge of resistance patterns for animal pathogenic bacteria in Denmark, as no official surveillance of this is in place. In this study, we present current knowledge of such resistance levels for some of the most important pig pathogenic bacteria, collected during the period 2004–2017. The findings are discussed and compared to patterns in antibiotic prescription for pigs.

## Results

Figures were aggregated to represent the periods 2004–2007, 2008–2011, 2012–2015, 2016, and 2017, respectively. For 2016 and 2017, only resistance levels for *A. pleuropneumoniae*, *E. coli*, and *S. suis* were included. The results from 4 years were combined to obtain more robust data, as the annual number of isolates for some of the bacteria were low.

For *E. coli*, isolates were with few exceptions susceptible to fluoroquinolones and colistin during all periods, whereas high occurrence of resistance was recorded for ampicillin, spectinomycin, streptomycin, sulfonamide, and tetracycline. MIC distributions and percent resistance are shown in Table 1A-E. Roughly, 7 out of 10 isolates were resistant to streptomycin, sulfonamide, and tetracycline. In 2017, one out of 72 *E. coli* O149 isolates was resistant to both ceftiofur and cefotaxime, suggesting that cephalosporin resistance is low, but not entirely absent. Resistance to neomycin decreased from 31.3% of the isolates in 2004–2007 to 14.7% in 2008–2011 and again to 9.6% in 2012–2015. In 2016 and 2017, neomycin resistance re-emerged to 11.9 and 13.9% respectively (Table 1D-E, Table 2). These changes are statistically significant and were also reflected in changes in MIC_90_. In contrast, the resistance to florfenicol increased steadily from 2.1% in 2004–2007 to 3.4% in 2008–2011, 5.2% in 2012–2015, 11.9% in 2016, and finally 18.1% in 2017 (Table 1A-E). This increase was also reflected in a change in MIC_90_ but not in MIC_50_. Data from VetStat on consumption of neomycin and florfenicol are shown in Table 2 together with resistance data. There was a clear temporal connection between usage and resistance for these compounds. A statistically significant increase in resistance was observed for trimethoprim (*p* < 0.00023), although it did not cause changes in MIC_50_ or MIC_90_ (Table 1). During 2016 and 2017, resistance to nalidixic acid increased to 10.2 and 19.4%, which is significantly higher compared to figures from 2004 to 2015. The isolates in 2016 and 2017 that were resistant to nalidixic acid had elevated MIC values for ciprofloxacin, although they were still categorized as sensitive. For other compounds, no major developments in resistance occurred during the observation period.

**Table 1:** Distribution of MIC values and occurrence of resistance in *E. coli* from pigs

Antimicrobial agent	% Resistant	Distribution (isolates) of MICs																				
		0.015	0.03	0.06	0.125	0.25	0.5	1	2	4	8	16	32	64	128	256	512	1024	2048	>2048	MIC50	MIC90
A - Distribution of MICs and occurrence of resistance in haemolytic *E. coli* from pigs (n=978), 2004-2007																						
Tetracycline	73.3								255	3	3	9	197	511							>32	>32
Chloramphenicol	16.5								94	594	116	13	60	13	88						4	64
Florfenicol	2.1								251	588	118	19	2								4	8
Ampicillin	42.1							186	315	52	9	4	5	407							2	>32
Amoxicillin + clavulanic acid	1.2								417	231	296	22	9	3							4	8
Trimethoprim	37.5									610	1	1		366							4	>32
Sulfonamide	68.3													273	5	32	86	84	498		>1024	>1024
Streptomycin	69.8									162	73	60	121	161	401						64	>64
Gentamicin	8.1							886	5	8	33	32	9	5							≤1	≤1
Neomycin	31.3								660	9	3	31	89	186							≤2	>32
Apramycin	8.2									858	37	3	1	25	54						≤4	8
Ciprofloxacin	0.5		886	16	21	44	6			2	3										≤0.03	≤0.03
Nalidixic acid	0.0									369	527	3	6	23	50						8	8
Colistin										967	4		1		6						≤4	≤4
Spectinomycin	0.0											360	69	66	107	251	125				64	>256
B - Distribution of MICs and occurrence of resistance in haemolytic *E. coli* from pigs (n=693), 2008-2011																						
Tetracycline	68.8								200	7	9	3	92	382							>32	>32
Chloramphenicol	14.0								35	401	150	10	46	17	34						4	32
Florfenicol	3.4								92	461	116	17	3		4						4	8
Ampicillin	49.2							154	182	16		1	2	338							4	>32
Amoxicillin + clavulanic acid	1.3								283	150	228	23	9								4	8
Trimethoprim	47.2							333	3	30			1	326							4	>32
Sulfonamide	67.4													217	8	1		5	462		>1024	>1024
Streptomycin	71.9										160	35	69	76	114	239					128	>128
Gentamicin	7.4						448	178	15	1	8	17	26								≤0.5	1
Neomycin	14.7								565	19	6	9	16	77							≤2	>32
Apramycin	6.9									531	105	9	1	47							≤4	8
Ciprofloxacin	0.1	492	160	9	1	24	3	1	2	1											≤0.15	0.03
Nalidixic acid	4.8									653	5	2	2	11	20						≤4	≤4
Colistin	7.0							637	5	49											≤1	≤1
Spectinomycin	51.8											233	43	58	50	90	219				128	>256
C - Distribution of MICs and occurrence of resistance in haemolytic *E. coli* from pigs (n=1026), 2012-2015																						
Tetracycline	69.0								298	10	10	6	77	625							>32	>32
Chloramphenicol	13.9								38	581	242	22	56	39	48						4	32
Florfenicol	5.2								92	688	193	18	4	4	27						4	8
Ampicillin	48.3							256	232	39	1	2	4	492							4	>32
Amoxicillin + clavulanic acid	2.0								437	190	335	43	21								4	8
Trimethoprim	46.2							542	4	2	4		1	473							≤1	>32
Sulfonamide	67.4													321	13		2	8	682		>1024	>1024
Streptomycin	70.9										243	55	58	85	110	475					128	>128
Gentamicin	8.8						657	254	23	2	11	34	45								≤0.5	2
Neomycin	9.6								874	44	9	5	16	78							≤2	8
Apramycin	7.8									810	129	7		80							≤4	8
Ciprofloxacin	0.1	468	317	19	185	24	10	2			1										0.03	0.125
Nalidixic acid	3.4									980	8	3	1	10	24						≤4	≤4
Colistin	1.8							1002	6	7	7	1	3								≤1	≤1
Spectinomycin	50.2											328	100	83	54	103	358				128	>256
D - Distribution of MICs and occurrence of resistance in haemolytic *E. coli* O149 from pigs (n=59), 2016																						
Tetracycline	67.8								17	2			40								>32	>32
Chloramphenicol	25.4								2	39	3		3	12							4	64
Florfenicol	11.9								7	40	5	1	1	5							4	8
Ampicillin	50.8							8	19	2			30								32	>32
Cefotaxime	1.7					58				2											≤0.25	≤0.25
Trimethoprim	47.5							31				1	27								≤1	32
Sulfonamide	69.9													18				41			>1024	>1024
Streptomycin	67.8										16	2	7	9	24						64	128
Gentamicin	6.8						42	12	1			4									≤0.5	1
Neomycin	11.9								49	3			7								≤2	32
Apramycin	6.8									53	2		4								≤4	8
Ciprofloxacin	0.0	49	4			5		1													≤0.015	0.25
Nalidixic acid	10.2									53				6							≤4	64
Colistin	0.0							59													≤1	≤1
Spectinomycin	42.4											27	4	3	4	21					32	256
E - Distribution of MICs and occurrence of resistance in haemolytic *E. coli* O149 from pigs (n=72), 2017																						
Tetracycline	62.5								26	1			2	43							>32	>32
Chloramphenicol	23.6									36	17	2	3	1	13						4	>64
Florfenicol	18.1								3	37	19				13						4	>64
Ampicillin	45.8							5	31	3			1	32							2	>64
Cefotaxime	2.8				70						2										≤0.25	≤0.25
Trimethoprim	55.6							32						40							>32	>32
Sulfonamide	61.1													28				1	43		>1024	>1024
Streptomycin	77.8										15	1	12	6	12	26					128	>128
Gentamicin	9.8						58	7			3	3	1								≤0.5	1
Neomycin	13.9								62				1	9							≤2	>64
Apramycin	9.8									63	2		7								≤4	8
Ciprofloxacin	0.0	50	7	1		11	3														≤0.15	0.25
Nalidixic acid	19.4									56	2			14							≤4	64
Colistin	1.4							71			1										≤1	≤1
Spectinomycin	59.9											27	1	3		15	26				256	>256
Ceftiofur	1.4						70			1		1									≤0.5	≤0.5

**Table 2:** Usage of florfenicol and neomycin (kg active compound) for pigs, and antimicrobial resistance (% resistant isolates) to florfenicol and neomycin among *E. coli* from Danish pigs, 2001–2017

Compound	Year	2001	2002	2003	2004	2005	2006	2007	2008	2009	2010	2011	2012	2013	2014	2015	2016	2017
Florfenicol	kg	1	< 4	75	97	79	65	62	83.6	121	150	226	164	263	244	338	321	381
	% R	n.d	n.d.	n.d	2.1 Average of 4 years				3.4 Average of 4 years				5.2 Average of 4 years				11.9	18.1
Neomycin	Kg	n.d	n.d	n.d	4616	4259	4206	2163	149	177	156	156	163	35	0	0	0	2283
	% R	n.d.	n.d.	n.d.	31.3				14.7				9.6				11.9	13.9

% R: Percent resistant isolates

There were differences between *E. coli* serovars. Serovar O149 and O138 had similar resistance patterns while O139 was less resistant to most compounds, i.e. to ampicillin, chloramphenicol, florfenicol, gentamicin, nalidixic acid, neomycin, sulphonamides, spectinomycin, streptomycin, tetracycline and trimethoprim (Table 3).

**Table 3:** Comparison of resistance in *E. coli* serovars O138, O139, and O149 from 2016 to 2017

Compound	*E. coli* O138 *N* = 19 R (%)	*E. coli* O139 *N* = 76 R (%)	*E. coli* O149 *N* = 132 R (%)
Ampicillin	13 (68)	29 (38)	64 (48)
Amoxicillin-clavulanic acid	1 (5)	0 (0)	3 (2)
Apramycin	3 (16)	7 (9)	12 (9)
Ceftiofur	0 (0)	0 (0)	2 (2)
Chloramphenicol	4 (21)	7 (9)	33 (25)
Ciprofloxacin	0 (0)	0 (0)	0 (0)
Colistin	0 (0)	0 (0)	0 (0)
Florfenicol	1 (5)	3 (4)	20 (15)
Cefotaxime	1 (5)	0 (0)	3 (2)
Gentamicin	3 (16)	3 (4)	9 (7)
Nalidixic acid	0 (0)	0 (0)	20 (15)
Neomycin	2 (11)	0 (0)	17 (13)
Sulphamethoxazole	15 (79)	40 (53)	86 (65)
Spectinomycin	12 (63)	24 (32)	67 (51)
Streptomycin	14 (74)	39 (51)	97 (73)
Tetracycline	15 (79)	43 (57)	85 (64)
Trimethoprim	11 (58)	31 (41)	69 (52)

The *A. pleuropneumoniae* isolates had high MIC values for erythromycin but with few exceptions susceptible to all other antimicrobial agents tested, including other macrolides, tulathromycin and tilmicosin. A small proportion of isolates was resistant to tetracyclines displaying a bimodal MIC distribution of the isolates. MIC distributions and percent resistance are shown in Additional file 2: Table S2A-E. No statistically significant differences in resistance were observed between periods except for a minor but significant increase in resistance to tetracycline from 4.0% in 2004–2007 to 7.6% in 2008–2011 and 2012–2015 (Additional file 2: Table S2). MIC distribution for tetracycline was clearly bimodal in a resistant and a sensitive group. A few isolates showed resistance to ampicillin.

The majority of the isolates belonged to the serotypes O2 and O6, but there were no significant differences in resistance patterns between serotypes (data not shown).

For *S. suis* MIC distributions and percent resistance are shown in Table 4A-E. High levels of resistance were recorded to tetracycline, around 75% throughout the whole period 2004–2017. For erythromycin, tiamulin, and trimethoprim an increasing trend was observed. A wide range of MIC values to tiamulin were recorded for *S. suis*, most isolates in the range of 0.5–2 μg/ml, however, the proportion of isolates with high MIC values increased over time. This was also reflected in an increase in both MIC_50_ and MIC_90_. Tiamulin is the 3rd most frequently used antimicrobial in pigs, after tetracyclines and macrolides. The resistance level for erythromycin increased considerably from 26.1% in 2004–2007 to 48.0% in 2017. For trimethoprim the increase was also pronounced from 1.8% in 2004–2007 to 23.0% in 2017, and MIC_90_ increased from ≤1 to 8 μg/ml. No other major developments were observed during the period 2004–2017. Both MIC_50_ and MIC_90_ for penicillin were low but a few isolates had MIC values above the clinical breakpoint. For tetracycline, sulphonamides, trimethoprim, erythromycin, streptomycin, spectinomycin, and tiamulin, bimodal MIC distributions occurred.

**Table 4:** Distribution of MIC values and occurrence of resistance in S. suis from Danish pigs

Antimicrobial agent	% Resistant	Distribution (number of isolates) of MICs																
		0.06	0.125	0.25	0.5	1	2	4	8	16	32	64	128	256	512	>512	MIC50	MIC90
A - Distribution of MICs and occurrence of resistance in *S. suis* (n=448) from pigs, 2004-2007																		
Tetracycline	79.5				43	49	58	77	9	6	46	160					4	>32
Chloramphenicol	0.2						100	318	29		1						4	4
Florfenicol	0.9					163	277	4	2	2							2	2
Penicillin	1.3	427	6	5	4	4	2										≤0.06	≤0.06
Ceftiofur	0.2		423	10	6	5	2	1	1								≤0.125	≤0.125
Trimethoprim	1.8					425	4	2	5		1	7					≤1	≤1
Sulfametoxazol											281	10	2		3	152	≤32	>512
Sulfa-trimethoprim	1.1			435	6	2		1		4							≤0.25	≤0.25
Erythromycin	26.1			326	5	3	5	2	3	1	103						≤0.25	>16
Streptomycin							5	21	137	134	53	30	68				16	>64
Ciprofloxacin	4.9		27	129	225	43	21	1									0.5	1
Spectinomycin										358	42	2	4	4	38		≤16	64
Tiamulin				48	67	177	100	6	4	6	28	12					1	8
B - Distribution of MICs and occurrence of resistance in *S. suis* (n=331) from pigs, 2008-2011																		
Tetracycline	72.2				49	43	31	51	7	4	32	114					4	>32
Chloramphenicol	0.6						61	240	28	2							4	4
Florfenicol	0.0					98	228	5									2	2
Penicillin	0.9	315	3	6	3	3											≤0.06	≤0.06
Ceftiofur	0.0		112	10	1	3	2										≤0.125	0.25
Trimethoprim	6.6				179	122	7	1	4	3	5	9					≤0.5	1
Sulfametoxazol											296	5	2		1	27	≤32	64
Sulfa-trimethoprim	0.0			323	6	2											≤0.25	≤0.25
Erythromycin	47.1			173	2	1	3	2	3	1	146						≤0.25	>16
Streptomycin								19	76	102	36	16	81				16	>64
Gentamicin				3	1	1	31	130	35	1	1						4	8
Ciprofloxacin	3.9		22	131	139	25	12	1									0.5	1
Spectinomycin										229	33	1	1	8	59		≤16	>256
Tiamulin				44	60	116	41	7	8	13	23	19					1	32
C - Distribution of MICs and occurrence of resistance in *S. suis* (n=400) from pigs, 2012-2015																		
Tetracycline	77.3				37	54	59	49	6	12	56	127					8	>32
Chloramphenicol	0.5						88	276	34	2							4	4
Florfenicol	0.3					140	252	7			1						2	2
Penicillin	1.8	370	8	5	9	4	2	1			1						≤0.06	≤0.06
Ceftiofur																		
Trimethoprim	14.8				320	14	7	23	2	3	4	27					≤0.5	4
Sulfametoxazol											300	26	18	5	3	48	≤32	>512
Sulfa-trimethoprim	1.0			368	17	5	6	2		2							≤0.25	≤0.25
Erythromycin	54.8			179	2	1	3	12	8		195						8	>16
Streptomycin								13	91	112	49	26	109				16	>64
Gentamicin				1	1	4	53	233	100	7	1						4	8
Ciprofloxacin	1.8		10	171	176	36	6	1									0.5	1
Spectinomycin										293	24	1	3	1	78		≤16	>256
Tiamulin				54	67	99	86	16	6	14	36	22					1	32
D - Distribution of MICs and occurrence of resistance in *S. suis* (n=151) from pigs, 2016																		
Tetracycline	73.5				10	30	23	13	1	2	27	45					4	>32
Chloramphenicol	0.7						27	117	6		1						4	4
Florfenicol	0.7					54	95	1		1							2	2
Penicillin	0.3	135	1	2	7	4	1										≤0.06	≤0.06
Trimethoprim	21.2				115	2	2	11	8		2	11					≤0.5	8
Sulfa-trimethoprim	4.6			118	6	3	15	3		4							≤0.25	2
Erythromycin	51.7			72		1		1	5	72							8	16
Streptomycin								14	46	30	15	5	41				16	>64
Spectinomycin										92	18	1		2	38		≤16	>256
Tiamulin				15	12	26	45	9	4	3	18	19					2	>32
E - Distribution of MICs and occurrence of resistance in *S. suis* (n=152) from pigs, 2017																		
Tetracycline	75.0				13	25	24	29	1	1	17	42					4	>32
Chloramphenicol	0.0						23	115	14								4	4
Florfenicol	0.0					28	112	2									2	2
Penicillin	2.6	136	4	6	2	4											≤0.06	0.125
Trimethoprim	23.0				114	1	1	13	7		2	13					≤0.5	8
Sulfa-trimethoprim	8.6			122	2	6	9	5	2	3	3						≤0.25	2
Erythromycin	48.0			77	1	1		1	1	2	69						≤0.25	>16
Streptomycin								14	40	26	25	5	42				16	>64
Spectinomycin										69	44			3	36		≤16	>256
Tiamulin				9	12	32	43	9	7	4	21	15					2	32

All *S. hyicus* isolates displayed sensitivity towards chloramphenicol, florfenicol, and ciprofloxacin. Notably, no isolates were found resistant to cefoxitin, suggesting that no methicillin resistant *S. hyicus* occurred. The highest resistance frequency was recorded for penicillin (82.2%) for which a very large range of MIC values were recorded from ≤0.06 to > 16 μg/ml and all values in between (Additional file 3: Table S3). High resistance levels were also found for tetracycline and tiamulin during the period 2004–2015. MIC distributions and percent resistance are shown in Additional file 3: Table 3A-C. Statistically significant increases in resistance were recorded in 2008–2011 for erythromycin (*p* < 0.0014), streptomycin (*p* < 0.01), and spectinomycin (*p* < 0.00022) compared to 2004–2007 figures, but also resistance to trimethoprim increased during the period from 2004 to 2015.

All *B. bronchiseptica* isolates were resistant to ampicillin and except for one isolate, sensitive to florfenicol. The MIC distributions for all tested compounds are shown in Additional file 4: Table S4. No major changes in distributions occurred during the period 2004–2017, but the numbers were low (Additional file 4: Table S4A-C).

## Discussion

In this study we present the latest available data on MIC values and sensitivity of important pathogenic bacteria in Danish pig production to an array of antibiotics. This is important both with respect to recommendations for treatment of infections in pigs and for human health due to occurrence of potential critical resistances. The present data have already formed the basis for the recent update of treatment guidelines for pigs in Denmark. The temporal changes in resistance we found for several bacteria to several antibiotics clearly show that resistance levels are not static and a continuous surveillance is therefore necessary.

A very high occurrence of resistance was found in *E. coli*. In the present study, the highest levels of resistance were observed for tetracycline and streptomycin, where approximately 70% isolates displayed resistance. Additionally, high resistance levels were observed for ampicillin, trimethoprim, sulfonamide, and spectinomycin. High resistance levels to these compounds in pathogenic isolates of *E. coli* have also been reported by other researchers [16–18]. A widespread occurrence of co-resistance to these antimicrobials is also reported from surveillance of commensal *E. coli* from many countries [19]. This high resistance to these compounds may be explained by a general high usage of these compounds combined with co-selection. Despite the restrictions on the use of quinolones in production animals that were enforced in 2002, we found resistance to nalidixic acid, albeit at low levels. Only few isolates were resistant to fluoroquinolones, but nalidixic acid resistant isolates had elevated MIC values to ciprofloxacin suggesting a mutation in the *gyrA* or *parC* gene [20]. From a one-health point of view, fluoroquinolones should not be used for treatment of animals as long as effective alternatives are available. The antimicrobial sensitivity of *E. coli* differs greatly from country to country, which likely reflects differences in usage. Thus, Hendriksen et al. [17] found the lowest levels of antimicrobial resistance in *E. coli* isolates from Norway, Sweden, and Finland, where usage is low, and high levels in countries such as Spain, Portugal, and Belgium, where usage is high. These authors found low resistance to ciprofloxacin with the notable exception of Spain and Portugal. Recent data from Sweden also showed that the highest resistance was to ampicillin, streptomycin, sulphonamides, trimethoprim, and tetracycline in isolates from diagnostic submissions (not serotyped), although at lower levels than in Denmark [21].

Resistance levels of *E. coli* were relatively stable over time to many antibiotics, but with notable exceptions. First, the resistance to florfenicol increased steadily from 2.1% in 2004 to 18.1% in 2017. This increase seems to reflect a usage increasing from almost zero in 2001 to the so far highest usage of 381 kg in 2017 (Table 2). Florfenicol is not registered for treatment of intestinal infections in Danish pigs but for respiratory infections, so the increase in resistance among *E. coli* isolates must have developed due to treatment of other diseases, i.e. respiratory infections, or due to co-selection. Another interesting development occurred for neomycin. Previously, neomycin was widely used for treatment of weaning diarrhea until 2008, but in recent years until 2017, colistin was recommended as first choice antimicrobial for intestinal infections in pigs. Neomycin for oral administration was taken off the market in 2008, and this has been followed by a decrease in resistance to neomycin (Table 2). However, after the emergence of *mcr1*-mediated resistance to colistin in many countries (although not Denmark), usage of colistin for pigs has almost entirely stopped from the beginning of 2017. Neomycin usage has therefore increased since a new product for oral administration was introduced on the market in 2017, and in 2017 resistance to neomycin seems to be increasing. Over the coming years, we will see whether this increase is a trend or merely random fluctuations.

In general, resistance levels were considerably lower among *E. coli* serovar O139 isolates compared to O149 and O138, suggesting significant differences between serovars (Table 3). The reason for this difference is currently unknown but may relate to differences in disease patterns and therefore treatment procedures: O149 and O138 cause diarrhea and therefore receive the same treatment, whereas O139 causes oedema disease, which may be subject to other treatment procedures. In general, higher resistance levels are observed in virulent, clinical isolates from diseased pigs compared to isolates from healthy pigs, which are presumably mostly commensal isolates [17], and lower levels of resistance have been reported in *E. coli* from organic pigs compared to conventional [22]. Both observations most likely reflect the differences in exposure to antimicrobials. Many reports on antimicrobial resistance in *E. coli* from pigs do not mention the serotype, and therefore they do not take into account that there may be these differences.

In this study, high MIC values for *A. pleuropneumoniae* were recorded for erythromycin, whereas all isolates were susceptible to newer macrolide drugs, tulathromycin and tilmicosin, which together with tildipirosin are registered and widely used for treatment of respiratory tract infections in pigs. We have no data for tylosin but the literature suggests that there can be some variability in sensitivity. In a study of 95 isolates [23] 6 isolates had an MIC value of 1 μg/ml, 69 had an MIC value of 2 μg/ml, whereas the remaining 20 isolates had an MIC > 32 μg/ml. This suggested a clear distinction between wildtype and resistant isolates, the majority being wildtype.

Apart from erythromycin, *A. pleuropneumoniae* isolates showed full sensitivity or low levels of resistance to other antimicrobial compounds tested. Similar observations were obtained for isolates from Poland, The Netherlands, France and England incl. Wales, but with notable differences: isolates from England tended to display considerably more resistance to tetracycline (22–37%) and trimethoprim-sulfonamide (13–46%), and isolates from England and Poland had considerably higher resistance to ampicillin (2–7 and 8%, respectively) [17]. In an Australian investigation by Dayao et al. [24] resistance to penicillin (8.5%) was also noticed. In a large study of isolates from Canada and the USA from 2011 to 2015 [25] approximately 10–15% of the *A. pleuropneumoniae* isolates were resistant to ampicillin with MIC values ≥16 μg/ml, which is far higher than the values we found in this study. Sweeney et al. [25] reported high resistance to tetracyclines, almost 100%, and with most of the isolates with MIC ≥8 μg/ml. We found a much lower resistance and also considerably lower MIC values for the majority of isolates (Additional file 2: Table S2A-E). Very high levels of resistance to tetracyclines (73.8%) were also reported from Spain [2], the Czech Republic (23.9%) [26], and Italy (17.2–70%) [27], and in the study by Gutiérrez-Martín et al. [2] the resistance to tetracyclines was increasing over time. We noticed some fluctuation in resistance to tetracyclines in Denmark but no increasing tendency. In the Italian study by Vanni et al. [27], also very high resistance was found to penicillins and macrolides, including tilmicosin and tulathromycin. Obviously, there seem to be marked differences between countries, which are not merely reflections of differences in choice of breakpoints. Although the association may not be direct, it is likely to be linked to overall usage and treatment patterns, e.g. dosage and treatment periods, as many of the major meat producing countries in Europe have a far higher use of tetracyclines than Denmark [7]. Even though tetracyclines constitute the most frequently used antibiotic class for Danish pigs, macrolides and pleuromutilins are almost as frequently used. In contrast, in many other European countries, tetracyclines have comprised the vast majority of antimicrobials used in meat production [7]. Broad-spectrum penicillins (mostly amoxicillin) is the 4th most frequently used compound group in Denmark [14, 15]. However, the most commonly prescribed drugs for treatment of porcine respiratory tract infections are tetracyclines, pluromutilins, macrolides, and penicillins [14, 15, 28]. Overall, there are still good opportunities to treat infections by *A. pleuropneumoniae* with antibiotics, but the emergence of strains resistant to penicillins and modern macrolides in some countries is very worrying, as it may ultimately leave fluoroquinolones or cephalosporins as some of the only options for treatment of outbreaks of pleuropneumonia in pigs. It underpins the importance of prudent use of antimicrobials and use of vaccines and biosecurity measures to prevent outbreaks. In addition, the increasing resistance to some of the most commonly used antimicrobials stresses that proper diagnostics and sensitivity testing should be performed at each outbreak.

In this study, approximately 75% of all *S. suis* isolates were found to be resistant to tetracycline and with increasing resistance to erythromycin and trimethoprim. For other compounds, resistance was low. In a recent study of *S. suis* from pigs in different European countries, the highest occurrence of resistance in Denmark was recorded for tetracycline (52.2%), followed by trimethoprim (51.5%) in 2003 [17]. Some variations in the sensitivity pattern were observed between the different countries. In general, a high occurrence of tetracycline resistance (48 to 92%) was found in France, England, The Netherlands, Poland and Portugal. Essentially all *S. suis* isolates were found to be sensitive to penicillin. However, 8.1% of the isolates were resistant to penicillin in Poland and 13% of the isolates were resistant to penicillin in Portugal [17]. In the present study, only few isolates were resistant to penicillin and there was no indication of any increasing tendency. The recorded resistance to penicillin reported from Poland and Portugal is concerning, since penicillin resistance in streptococci is uncommon. Furthermore, penicillin is the recommended first choice for treatment of streptococcal infections by the Danish Veterinary and Food Administration. Penicillin resistance was also reported from Canada and the USA by Sweeney et al. [25] who found 16–26.4% *S. suis* resistant to penicillin. These authors also found most isolates resistant to tetracycline and having very high MIC values to macrolides.

It also seemed that MIC50 and MIC90 for tiamulin were increasing, suggesting lower susceptibility of *S. suis* to tiamulin. Tiamulin is the third most frequently used antimicrobial in pigs, after tetracyclines and macrolides, and generally used for treatment of *Brachyspira* and *Lawsonia* infections. Any shift in susceptibility of *S. suis* must therefore likely be ascribed to selection due to treatment of other infections.

Among the *S. hyicus* isolates, resistance was recorded to a wide range of antimicrobial agents in the panel. Penicillin resistance was found in almost nine out of ten isolates in this study. Additionally, high resistance levels were found to macrolides, tetracycline, sulfonamides and streptomycin. The results conducted in this study are supported by previous reports from Denmark [29–31]. In this study, all *S. hyicus* isolates were found sensitive to ciprofloxacin, chloramphenicol and florfenicol. The resistance level for *S. hyicus* was monitored by the DANMAP program in 2003, revealing a significant increase in penicillin resistance from 54% in 2000 to 84% in 2003, however the number of isolates was low. Findings in this study demonstrate that the resistance level for penicillin essentially have remained unchanged and high since 2003, except for some fluctuations. Results from Germany revealed high occurrences of antimicrobial resistance in *S. hyicus* to sulfonamides and tetracycline [16], which are in accordance to the data provided in this study. However, many available international publications are old and may not be valid at present time. Outbreaks of disease caused by *S. hyicus* are no longer frequent in Denmark and consequently, treatment is rarely required. Autogenous vaccines are used to some extent. The high resistance to penicillin must therefore be ascribed to selection after exposure to beta-lactam antibiotics for treatment of other diseases.

For *B. bronchiseptica*, there is a lack of approved clinical breakpoints. Using the breakpoint of ≥2 μg/ml for ampicillin, all isolates were resistant, which is in accordance with other reports [24, 32]. The MIC distributions for most compounds showed a unimodal distribution, which is also what Prüller et al. [32] reported, but MIC values for e.g. streptomycin, spectinomycin and sulphonamides were very high. Notable exception was tetracycline, for which there was a clearly bimodal distribution, suggesting a sensitive and a resistant population. This was also reported by Prüller et al. [32]. In a German study, Kadlec et al. [33] reported low frequency of acquired resistance to ampicillin, chloramphenicol and tetracycline, while Eun-Kyung et al. [34] reported all isolates of *B. bronchiseptica* to be sensitive to neomycin, amoxicillin, and gentamicin and 92.7% of the isolates were susceptible to ciprofloxacin. Care should be taken comparing these results, as they may partly be due to differences in choice of breakpoints, in particular concerning amoxicillin. This emphasizes the importance of establishing approved clinical breakpoints. *Bordetella bronchiseptica* has been described to be intrinsically resistant to ampicillin due to production of beta-lactamases [32, 35]. In general, *B. bronchiseptica* causes a mild or non-progressive inflammation in the nasal cavity that passes by spontaneously and usually needs no treatment on its own. However, if the bacterium is co-infecting with toxigenic *Pasteurella multocida,* it can lead to severe progressive atrophic rhinitis [5]. Further, in some cases *B. bronchiseptica* causes pneumonia in young piglets. Hence, it is of importance that we continue to monitor the resistance trends for this bacterium. In veterinary medicine, tetracyclines are often used to manage diseases caused by *B. bronchiseptica.* Speakman et al. [36] described a plasmid-encoded tetracycline resistance gene, *tetC*, but in our study the vast majority of isolates had MIC values ≤2 μg/ml for tetracycline, which should probably be considered sensitive. In Denmark, macrolides (mainly tylosin) are also often used against *B. bronchiseptica*. Dayao et al. [24] reported no resistance to tulathromycin, but unfortunately, we have no data on Danish isolates because tulathromycin and tylosin are not included in the currently used test panel. However, this is under revision and treatment should always be based on a sensitivity test.

In EU, a surveillance has been established on the prevalence of resistance in human and zoonotic pathogens and commensal indicator bacteria, whereas less effort is put on veterinary pathogens. Existing data for both human and veterinary pathogens reveal substantial geographic variations in the resistance trends to different classes of antimicrobial compounds in Europe and worldwide [37]. However, for some pathogens and antimicrobials limited data are available, thus it is very important to continue the surveillance of antimicrobial resistance for the major pathogens causing infectious diseases in human health-care settings and in veterinary medicine. Comparison of existing data from multiple laboratories is also hampered by inconsistencies in methodology, selection of antimicrobial substances in the test panel, variations in interpretation criteria for clinical breakpoints, etc. Therefore, comparison of data must be made with caution. Antimicrobial sensitivity testing is used to provide information concerning the efficacy of antimicrobial agents and thus determine whether an antibiotic is suitable to treat a specific condition, and it can only be recommended to use sensitivity testing more often prior to treatment. Furthermore, sensitivity testing of antimicrobial drugs is challenging and requires a uniform standard method and approved breakpoints in order to determine whether an isolate is sensitive, intermediate or resistant. Unfortunately, approved clinical breakpoints are available only for a very limited number of drug-bug combinations and much more effort is needed to establish breakpoints for the most commonly used antimicrobial agents in humans as well as animals. In this study, resistance data are presented as distributions of MICs, which allow each individual to interpret the results themselves by the usage of alternative sensitivity breakpoints.

## Conclusion

The obtained resistance patterns vary markedly between pathogens. However, within the individual pathogen the resistance pattern was relatively stable, with some fluctuations but generally without any major changes throughout the study period from 2004 to 2017. Notable exceptions were resistance to neomycin and florfenicol in *E. coli*. In general, low resistance levels were observed to the majority of the antimicrobial agents tested for *A. pleuropneumoniae*. In contrast, *E. coli* showed resistance to multiple compounds, while resistance to flouroquinolons, cephalosporins, and colistin was low. *Staphylococcus hyicus* showed high resistance to penicillin, tetracycline and macrolides whereas almost all isolates of *S. suis* were found to be sensitive to penicillin. Increasing resistance over the years was recorded for *S. suis* to erythromycin, tiamulin and trimethoprim. Changes in resistance patterns over time emphasize the need of continuous monitoring and adjustment of treatment recommendations. Likewise, the results emphasize the importance of sensitivity testing for correct treatment and optimization of responsible antimicrobial use. The study also pinpoint the need for establishment of standardized protocols and breakpoints in order to follow the development and give insight into the epidemiology of resistance.

## Methods

### Bacterial isolates and culturing conditions

A total number of 1966 *A. pleuropneumoniae*, 266 *B. bronchiseptica,* 2923 *E. coli*, 168 *S. hyicus*, and 1482 *S. suis* isolates, isolated from Danish pigs during the 14-year period from 2004 to 2017 were included in this study. All bacterial isolates were obtained from clinical samples submitted to The National Veterinary Institute, DTU, or to SEGES Laboratory for Pig Diseases in Kjellerup. The bacterial isolates were recovered by conventional culturing methods and identified by standard biochemical methods or matrix-assisted laser desorption/ionization-time of flight mass spectrometry (MALDI-TOF) as previously described [38]. Serotyping of *E. coli* and *A. pleuropneumoniae* was performed using slide agglutination.

### Antimicrobial sensitivity testing

The minimum inhibitory concentration (MIC) of different antimicrobial compounds was determined for each bacterial isolate by the broth microdilution sensitivity testing method using a semi-automatic system (SensiTitre, Trek Diagnostic Systems Ltd., UK) in accordance with the recommendations presented by the Clinical and Laboratory Standards Institute [39]. As control strains were used *E. coli* ATCC 25922, *Staphylococcus aureus* ATCC 29213, *Enterococcus faecium* ATCC 29212, *Streptococcus pneumoniae* ATCC 49619, and *A. pleuropneumoniae* ATCC 27090 [39].

The antimicrobials tested in this study included apramycin, cefotaxime, cefoxitin, ceftiofur, chloramphenicol, ciprofloxacin, colistin, erythromycin, florfenicol, gentamicin, nalidixic acid, neomycin, penicillin, spectinomycin, streptomycin, sulfa-TMP, sulfamethoxazol, tetracycline, tiamulin, tilmicosin, trimethoprim and tulathromycin. Different bacterial species were tested for different panels of antimicrobial agents. Three different MIC panels were used, which were custom made to represent both commonly used compounds for treatment and compounds relevant for surveillance of critical resistance. The compounds tested and the concentration ranges are indicated in the tables for each bacterium.

The results of the sensitivity tests are presented as MIC distributions. Clinical breakpoints from CLSI were used when available [39–41] and otherwise EUCAST clinical breakpoints or epidemiological cut-off values (www.EUCAST.org). The breakpoints used and references to where they were adopted from are shown in Additional file 1: Table S1. They are also indicated in each table. The resistance level was considered low at levels < 10% and high at levels > 40%.

Comparisons of resistance levels between years for each bacterial species were performed by a Chi-Square Test. Results were considered statistically significant when *p* < 0.05.

## Supplementary information

**Additional file 1: Table S1.** Antibiotics tested and the used breakpoint values (μg/mL) for *Escherichia coli*, *Streptococcus suis, Actinobacillus pleuropneumoniae*, *Staphylococcus hyicus* and *Bordetella bronchiseptica* from Danish pigs.

**Additional file 2: Table S2.** Distribution of MIC values and occurrences of resistance in *A. pleuropneumoniae* from Danish pigs

**Additional file 3: Table S3.** Distribution of MIC values and occurrences of resistance in *S. hyicus* from Danish pigs.

**Additional file 4: Table S4.** Distribution of MIC values and occurrences of resistance in *B. bronchiseptica* from Danish pigs

## Data Availability

The datasets used and/or analyzed during the current study are available from corresponding author on reasonable request.

## Abbreviations

CLSI: Clinical and Laboratory Standards Institute
MALDI-TOF: Matrix-assisted laser desorption/ionization – time of flight
MIC: Minimum inhibitory concentration
