# Supplementary material for: Antibiotic resistance in porcine pathogenic bacteria and relation to antibiotic usage
Source: BMC Vet Res. 2019 Dec 11;15:449. doi: 10.1186/s12917-019-2162-8 (PMC6907208; doi:10.1186/s12917-019-2162-8)
Supplement: Supplementary file 1 — Additional file 1: Table S1. Antibiotics tested and the used breakpoint values (μg/mL) for Escherichia coli, Streptococcus suis, Actinobacillus pleuropneumoniae, Staphylococcus hyicus and Bordetella bronchiseptica from Danish pigs. [file 12917_2019_2162_MOESM1_ESM.docx]

Table S1. Antibiotics tested and the used breakpoint values (µg/mL) for *Escherichia coli*, *Streptococcus suis, Actinobacillus pleuropneumoniae*, *Staphylococcus hyicus* and *Bordetella bronchiseptica* from Danish pigs.

|  | *E. coli* | | | | *Staphylococcus hyicus* | | | | *Streptococcus suis* | | | | *Actinobacillus pleuropneumoniae* | | | | *Bordetella bronchiseptica* | | | |
| --- | --- | --- | --- | --- | --- | --- | --- | --- | --- | --- | --- | --- | --- | --- | --- | --- | --- | --- | --- | --- |
|  | S | I | R | Ref. | S | I | R | Ref. | S | I | R | Ref. | S | I | R | Ref. | S | I | R | Ref. |
| Amoxicillin with clavulanic acid (1:2) | ≤8/4 | 16/8 | ≥32/16 | ^1^ |  |  |  |  |  |  |  |  |  |  |  |  |  |  |  |  |
| Ampicillin | ≤8 | 16 | ≥32 | ^1^ |  |  |  |  |  |  |  |  | ≤0.5 | 1 | ≥2.0 | ^2^ | ≤0.5 | 1 | ≥2.0 | ^2^ |
| Apramycin | ≤16 |  | ≥32 | ^5^ |  |  |  |  |  |  |  |  |  |  |  |  |  |  |  |  |
| Cefotaxime | ≤1 | 2 | ≥4 | ^1^ |  |  |  |  |  |  |  |  |  |  |  |  |  |  |  |  |
| Cefoxitin |  |  |  |  | ≤4 |  | ≥8 | ^1*^ |  |  |  |  |  |  |  |  |  |  |  |  |
| Ceftiofur | ≤2 | 4 | ≥8 | ^2^ |  |  |  |  | ≤2 | 4 | ≥8 | 2 | ≤2 | 4 | ≥8 | ^2^ |  |  |  |  |
| Chloramphenicol | ≤8 | 16 | ≥32 | ^1^ | ≤8 | 16 | ≥32 | ^1^ | ≤4 | 8 | ≥16 | ^1^ |  |  |  |  |  |  |  |  |
| Ciprofloxacin | ≤1 | 2 | ≥4 | ^1^ | ≤1 | 2 | ≥4 | ^1*^ | ≤0.5 | 1 | ≥2 | ^2^ | ≤0.25 | 0.5 | ≥1 | ^2^ |  |  |  |  |
| Colistin | ≤2 |  | ≥4 | ^3^ |  |  |  |  |  |  |  |  |  |  |  |  |  |  |  |  |
| Erythromycin |  |  |  |  | ≤0.5 | 1-4 | ≥8 | ^1^ | ≤0.25 | 0.5 | ≥1 | ^1^ |  |  |  |  |  |  |  |  |
| Florfenicol | ≤4 | 8 | ≥16 | ^2^ |  |  | ≥32 | ^4*^ | ≤2 | 4 | ≥8 | ^2^ | ≤2 | 4 | ≥8 | ^2^ | ≤2 | 4 | ≥8 | ^2^ |
| Gentamicin | ≤2 | 4 | ≥8 | ^3^ | ≤4 | 8 | ≥16 | ^1^ | NA | NA | NA | ^3^ |  |  |  |  |  |  |  |  |
| Nalidixic acid | ≤16 |  | ≥32 | ^1^ |  |  |  |  |  |  |  |  |  |  |  |  |  |  |  |  |
| Neomycin |  |  | ≥16 | ^4^ |  |  |  |  |  |  |  |  |  |  |  |  |  |  |  |  |
| Penicillin |  |  |  |  | ≤0.12 |  | ≥0.25 | ^1^ | ≤0.25 | 0.5 | ≥1.00 | ^2^ |  |  |  |  |  |  |  |  |
| Spectinomycin |  |  | ≥128 | ^4^ |  |  | ≥128 | ^4*^ |  |  |  |  |  |  |  |  |  |  |  |  |
| Streptomycin |  |  | ≥32 | ^4^ |  |  | ≥32 | ^4*^ |  |  |  |  |  |  |  |  |  |  |  |  |
| Sulphamethoxazole | ≤256 |  | ≥512 | ^1^ | ≤256 |  | ≥512 | ^1^ |  |  |  |  |  |  |  |  |  |  |  |  |
| Tetracycline | ≤4 | 8 | ≥16 | ^1^ | ≤4 | 8 | ≥16 | ^1^ | ≤0.5 | 1 | ≥2 | ^2^ | ≤0.5 | 1 | ≥2 | ^2^ |  |  |  |  |
| Tiamulin |  |  |  |  |  |  | ≥32 | ^4*^ |  |  |  |  | ≤16 |  | ≥32 | ^2^ |  |  |  |  |
| Trimethoprim | ≤8 |  | ≥16 | ^1^ | ≤8 |  | ≥16 | ^1^ | ≤2 |  | ≥4 | ^3^ |  |  |  |  |  |  |  |  |
| Trimethoprim-sulphonamide | ≤2/38 |  | ≥4/76 | ^1^ | ≤2/38 |  | ≥4/76 | ^1^ | ≤0.5/9.5 | 1/19 – 2/38 | ≥4/76 | ^2^ |  |  |  |  |  |  |  |  |
| Tulathromycin |  |  |  |  |  |  |  |  |  |  |  |  | ≤64 |  | ≥128 | ^2^ |  |  |  |  |
| Tilmicosin |  |  |  |  |  |  |  |  |  |  |  |  | ≤16 |  | ≥32 | ^2^ |  |  |  |  |

^1^ CLSI M100, 2018, ^2^ CLSI VET08 4^th^ ed., 2018, ^3^ EUCAST (v 8.1 Breakpoint Tables), ^4^ EUCAST Epidemiological cut-off values (ECOFFs), ^5^DANMAP 2015 porcine.

S: susceptible, I: intermediate, R: resistant, Ref.: reference for breakpoint data. *Adopted from *Staphylococcus aureus*.
