# Supplementary material for: Antibiotic resistance in porcine pathogenic bacteria and relation to antibiotic usage
Source: BMC Vet Res. 2019 Dec 11;15:449. doi: 10.1186/s12917-019-2162-8 (PMC6907208; doi:10.1186/s12917-019-2162-8)
Supplement: Supplementary file 3 — Additional file 3: Table S3. Distribution of MIC values and occurrences of resistance in S. hyicus from Danish pigs. [file 12917_2019_2162_MOESM3_ESM.docx]

Table S3A-C: Distribution of MIC values and occurrences of resistance in *S. hyicus* from Danish pigs.

Table S3A-C shows the MIC distributions and percent resistance in *S. hyicus* during the three periodes from 2004-2007 (3A), 2008-2011 (3B), and 2012-2015 (3C), respectively. Vertical solid lines indicate microbiological breakpoint values for antimicrobial resistance (preferably CLSI); vertical dotted lines indicate breakpoints for intermediate sensitivity. White fields represent the range of dilutions tested. MIC values equal to or lower than the lowest concentration tested are presented as the lowest concentration. MIC values greater than the highest concentration in the range are presented as one dilution step above the range.
