# Supplementary material for: Antibiotic resistance in porcine pathogenic bacteria and relation to antibiotic usage
Source: BMC Vet Res. 2019 Dec 11;15:449. doi: 10.1186/s12917-019-2162-8 (PMC6907208; doi:10.1186/s12917-019-2162-8)
Supplement: Supplementary file 4 — Additional file 4: Table S4. Distribution of MIC values and occurrences of resistance in B. bronchiseptica from Danish pigs [file 12917_2019_2162_MOESM4_ESM.docx]

Table S4 A-C: Distribution of MIC values and occurrences of resistance in B. bronchiseptica from Danish pigs

Table S4A-C shows the MIC distributions and percent resistance in *B. bronchiseptica* during the three periodes from 2004-2007 (4A), 2008-2011 (4B), and 2012-2015 (4C), respectively. Vertical solid lines indicate microbiological breakpoint values for antimicrobial resistance (preferably CLSI). White fields represent the range of dilutions tested. MIC values equal to or lower than the lowest concentration tested are presented as the lowest concentration. MIC values greater than the highest concentration in the range are presented as one dilution step above the range.
